# Effective Control of Chronic γ-Herpesvirus Infection by Unconventional MHC Class Ia–Independent CD8 T Cells

**DOI:** 10.1371/journal.ppat.0020037

**Published:** 2006-05-19

**Authors:** Douglas C Braaten, James Scott McClellan, Ilhem Messaoudi, Scott A Tibbetts, Kelly B McClellan, Janko Nikolich-Zugich, Herbert W Virgin

**Affiliations:** 1 Department of Pathology and Immunology, Washington University School of Medicine, St. Louis, Missouri, United States of America; 2 Department of Molecular Microbiology, Washington University School of Medicine, St. Louis, Missouri, United States of America; 3 Department of Microbiology and Immunology, Oregon Health and Science University, Beaverton, Oregon, United States of America; University of California San Francisco, United States of America

## Abstract

Control of virus infection is mediated in part by major histocompatibility complex (MHC) Class Ia presentation of viral peptides to conventional CD8 T cells. Although important, the absolute requirement for MHC Class Ia–dependent CD8 T cells for control of chronic virus infection has not been formally demonstrated. We show here that mice lacking MHC Class Ia molecules (*K^b−/−^xD^b−/−^* mice) effectively control chronic *γ-herpesvirus 68* (γHV68) infection via a robust expansion of *β_2_-microglobulin (β_2_-m)*-dependent, but *CD1d*-independent, unconventional CD8 T cells. These unconventional CD8 T cells expressed: (1) CD8αβ and CD3, (2) cell surface molecules associated with conventional effector/memory CD8 T cells, (3) TCRαβ with a significant Vβ4, Vβ3, and Vβ10 bias, and (4) the key effector cytokine interferon-γ (IFNγ). Unconventional CD8 T cells utilized a diverse TCR repertoire, and CDR3 analysis suggests that some of that repertoire may be utilized even in the presence of conventional CD8 T cells. This is the first demonstration to our knowledge that *β_2_-m*–dependent, but *Class Ia*–independent, unconventional CD8 T cells can efficiently control chronic virus infection, implicating a role for *β_2_-n*–dependent non-classical MHC molecules in control of chronic viral infection. We speculate that similar unconventional CD8 T cells may be able to control of other chronic viral infections, especially when viruses evade immunity by inhibiting generation of Class Ia–restricted T cells.

## Synopsis

In this paper the authors identify a *β2-microglobulin*–dependent but major histocompatibility complex (MHC) *Class Ia*– and *CD1*-independent class of CD8 T cells that effectively control chronic γ-herpesvirus infection in mice. The important point that should be of general interest to the readers of *PLoS Pathogens* is that an effective CD8 T cell response develops during chronic infection of mice lacking MHC Class Ia molecules. Enormous efforts have gone into characterizing the role of conventional CD8 T cells that recognize viral peptides together with MHC Class Ia molecules during chronic viral infection, and many vaccine approaches focus solely on this response. This paper shows that additional types of CD8 T cells can operate during chronic infection, and that indeed, conventional MHC Class Ia–restricted T cells may be dispensable for control of chronic herpesvirus infection. The authors believe this is a fundamentally important point because it raises the question of whether unconventional CD8 T cells are important for control of other chronic viral infections such as infection with HIV, Hepatitis C virus, Hepatitis B virus, or human herpesviruses.

## Introduction

A defining characteristic of herpesviruses is their ability to persist for the life of the infected host by establishing latent infection after acute infection is cleared. Herpesviruses can reactivate from latency, generating new infectious virus that can either re-initiate lytic replication (a process termed herein “persistent replication,” to distinguish it from replication during acute infection) or spread to a new host. Many γ-herpesviruses, including the human pathogens Epstein-Barr virus (EBV) and Kaposi's sarcoma-associated herpesvirus (KSHV), and the murine pathogen γ-*herpesvirus 68* (γHV68), establish life-long latent infections within hematopoietic cells [1–6]. Consequently, chronic γ-herpesvirus infections are frequently associated with the development of B cell malignancies, especially in immunocompromised individuals [7–12]. γ-Herpesvirus-associated diseases are particularly common in immunocompromised hosts, a fact indicating that the immune system normally controls chronic γ-herpesvirus infection and thereby prevents disease [10,11,13–16].

Immune control of chronic γHV68 infection has been studied extensively as a model for defining viral and host mechanisms that are responsible for maintaining latency as a stable equilibrium between virus and host [15–28]. After either intranasal or intraperitoneal infection [1,23], cells that harbor latent γHV68 can be found in hematopoietic organs such as the bone marrow and spleen, and in body cavities such as the peritoneum [1–5,29]. Latent γHV68 infection of splenocytes and peritoneal cells has been extensively characterized in wild-type mice, and two forms of γHV68 latency have been observed [23,25,30,31]. An early form of latency occurs by 16 d after infection that is characterized by a high efficiency of reactivation from latency (i.e., frequency of reactivation per number of latently infected cells) in an ex vivo assay, with the majority of genome-bearing cells reactivating [23,30]. A second, long-term form of latency occurs 28–42 d after infection and is characterized by a lower efficiency of reactivation ex vivo, with approximately 10% of genome-bearing cells reactivating [23,25,30]. This long-term form of latency reflects a stable relationship between the virus and the host that is independent of both dose and route of virus infection [23], but which can be perturbed by immunodeficiency [15,25,30,31]. During this equilibrium phase of latency, sensitive assays can detect a very small amount of infectious virus in some mice, consistent with reactivation from latency and persistent replication [29].

The immune system normally controls latency and chronic infection at several levels, including immune-mediated decreases in the number of latently infected cells [17,19,20,25] and regulation of the efficiency of reactivation from latency [25]. Different components of the immune system have specific roles in regulating γHV68 latency at different sites; for example, the absence of interferon-γ (IFNγ) increases the efficiency of reactivation of latently infected peritoneal but not spleen cells [25,31]. In addition to regulating latency, the immune system normally prevents most persistent lytic replication, which flares in immunocompromised mice, especially those lacking CD8 or IFNγ [15,16,25,29,32–34]. In addition to its role in limiting persistent replication, IFNγ (as well as IFNαβ) regulates latent γHV68 gene expression in vivo, and IFNγ can directly inhibit the reactivation of γHV68 from latency [32,33]. Persistent replication requires genes such as the *v-bcl2* and *v-cyclin* that are not required for replication during acute infection, which indicates that persistent replication occurs by a distinct mechanism from that required for acute replication [31,35]. Persistent replication is likely critical for the maintenance of a constant pool of latently infected cells [27,34,36] by infection and establishment of latency in uninfected target cells.

A large body of evidence indicates that CD8 T cells are important for control of acute, latent, and persistent γHV68 infection. CD8 T cell responses directed against Class Ia–presented peptides derived from lytic cycle proteins are detectable as early as 10 d after infection [21,37]. These CD8 T cells actively cycle for many months after infection [22,37,38], which is consistent with a role for CD8 T cells in the control of persistent replication [25]. TCR transgenic CD8 T cells that recognize the SIINFEKL peptide of ovalbumin can control infection of a recombinant γHV68 virus that expresses ovalbumin [19]. Furthermore, CD8 T cells that recognize the latency-associated γHV68 antigen M2 control the early form of γHV68 latency [28,39]. These data demonstrate that both lytic and latent γHV68 antigens are presented by Class Ia on infected cells during chronic infection. These data are supported by published studies demonstrating that either CD8 deficiency or β_2_-microglobulin (β_2_-m) deficiency is associated with increased latency and persistent replication [25,40–42], and by studies showing that antibody-mediated depletion of CD8 T cells from wild-type mice results in increased latent infection [20,43].

That classical CD8 T cells are involved in control of γHV68 infection is consistent with a critical role for Class Ia molecules in presenting γHV68-encoded antigens; however, the requirement for Class Ia molecules for control of infection has not been formally tested. This is of interest because some published studies suggest that unconventional T cells are stimulated during γHV68 infection. For example, T cell hybridomas isolated from γHV68-infected mice can respond to γHV68-infected cells in the absence of TAP1 or β_2_-m [42,44]. However, the use of *β_2_-m^−/−^* mice and cells as models for Class Ia deficiency is complicated by the fact that β_2_-m is also an important accessory molecule for the cell surface expression of multiple non-classical major histocompatibility complex (MHC) Class Ib molecules, including CD1d, M3, FcRn, Qa-1, and HFE (reviewed in [45,46]). It has also been reported that *β_2_-m^−/−^* mice are not completely Class Ia deficient [47–49].

To circumvent these limitations, we evaluated γHV68 infection in Class Ia–deficient (Class Ia) mice *(K^b−/−^xD^b−/−^)* [50,51] and in mice deficient in both Class Ia and β_2_-m molecules *(K^b−/−^xD^b−/−^xβ_2_-m^−/−^)* [52]; *K^b−/−^xD^b−/−^xβ_2_-m^−/−^* mice lack expression of both Class Ia and β_2_-m–dependent Class Ib molecules. Because the non-classical Class Ib molecule CD1d has been implicated in control of herpesvirus infection ([53], reviewed in [54]), we also analyzed mice deficient in both Class Ia and CD1d *(K^b−/−^xD^b−/−^xCD1d^−/−^)*. We found that mice lacking Class Ia molecules mount a substantial effector CD8 T cell response that requires β_2_-m but not CD1d. These unconventional effector CD8 T cells effectively controlled latent γHV68 infection. These data suggest that unconventional T cells could play an important role in control of other chronic viral infections, with important implications for the study of antigens recognized by CD8 T cells during chronic infection and, especially, for viruses that evade immunity by inhibiting MHC Class Ia expression.

## Results

### Class Ia–Deficient Mice Effectively Control Acute γHV68 Infection

**Figure 1:**
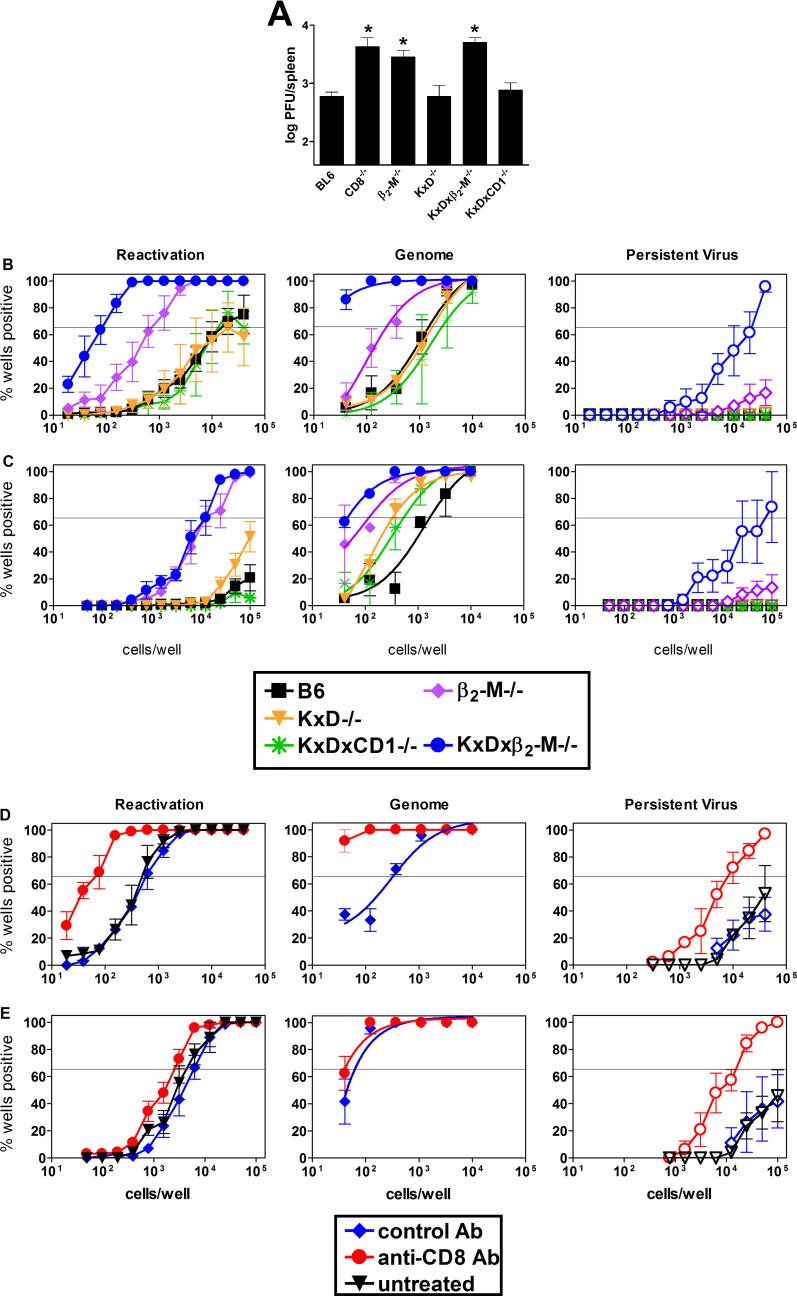
Class Ia–Deficient Mice Effectively Control γHV68 Infection in a β_2_-m– and CD8 T Cell–Dependent Manner (A) Log viral titers in spleen of B6, *CD8α^−/−^, β_2_-m^−/−^, K^b−/−^xD^b−/−^, K^b−/−^xD^b−/−^xCD1d^−/−^,* and *K^b−/−^xD^b−/−^xβ_2_-m^−/−^* mice 9 d after intraperitoneal infection with 10^6^ PFU of virus. Data were pooled from two experiments consisting of two to three mice each. An asterisk (*) indicates a significant increase in viral titer relative to B6 (*p* < 0.05). (B) Frequency of cells reactivating from latency ex vivo (left), frequency of cells bearing viral genome (middle), and persistent replication (right, open symbols) at day 42 post-infection in peritoneal cells from B6, β_2_-*m^−/−^, K^b−/−^xD^b−/−^, K^b−/−^xD^b−/−^xCD1d^−/−^,* and *K^b−/−^xD^b−/−^xβ_2_-m^−/−^* mice. On the *y*-axis is the percentage of wells positive for viral cytopathic effect (left and right) or viral genome (middle). The horizontal line within the graph indicates the 63.2% Poisson distribution line used to calculate the frequency of cells reactivating virus. For each group, cells were pooled from three to five mice. Data are the mean of three to four independent experiments ± SEM. (C) Frequency of latent infection in splenocytes from B6, β_2_-*m^−/−^, K^b−/−^xD^b−/−^, K^b−/−^xD^b−/−^xCD1d^−/−^,* and *K^b−/−^xD^b−/−^xβ_2_-m^−/−^* mice. For each group, cells were pooled from three to five mice. Data are the mean of three to four independent experiments ± SEM. (D) γHV68 latency at day 16 post-infection in peritoneal cells from *K^b−/−^xD^b−/−^xCD1d^−/−^* mice either untreated, treated with a control antibody (Ab), or depleted of CD8 T cells beginning 1 d prior to infection. Left: ex vivo reactivation of peritoneal cells from control-treated and CD8-depleted groups. Middle: frequency of peritoneal cells that contain viral genome in control-treated and CD8-depleted groups. Right: persistent replication in peritoneal cells from control-treated and CD8-depleted groups. (E) γHV68 latency at day 16 post-infection in splenocytes from *K^b−/−^xD^b−/−^xCD1d^−/−^* mice either untreated, treated with a control antibody, or depleted of CD8 T cells beginning 1 d prior to infection. For each group in D and E, cells were pooled from three to five mice. Data are the mean of three independent experiments ± SEM.

To begin to evaluate the role of Class Ia molecules during γHV68 infection, acute virus replication was compared in strains of mice 9 d after intraperitoneal injection of 10^6^ plaque forming units (PFU) of wild-type γHV68. Spleens from B6, *CD8^−/−^, β_2_-m^−/−^, K^b−/−^xD^b−/−^, K^b−/−^xD^b−/−^xβ_2_-m^−/−^,* and *K^b−/−^xD^b−/−^xCD1d^−/−^* mice were harvested, and the titer of virus present was determined (Figure 1A, Table 1). There were no significant differences in titers in the spleens from wild-type, *K^b−/−^xD^b−/−^,* and *K^b−/−^xD^b−/−^xCD1d^−/−^* mice, whereas mice lacking either CD8 or β_2_-m demonstrated splenic titers that were 8.7-fold (*p* = 0.03) and 5.1-fold (*p* = 0.04) higher, respectively, as compared to wild-type mice. We noted that *K^b−/−^xD^b−/−^xβ_2_-m^−/−^* mice failed to control infection compared to either *K^b−/−^xD^b−/−^* or wild-type mice. Furthermore, lack of CD8 resulted in decreased control of γHV68 replication even though lack of Class Ia molecules did not. Although there are several potential explanations for these findings, these data could be explained if a β_2_-m–dependent, but Class Ia–independent, population of CD8 T cells was important for the control of infection observed in the K^b−/−^xD^b−/−^ mice.

**Table 1:**
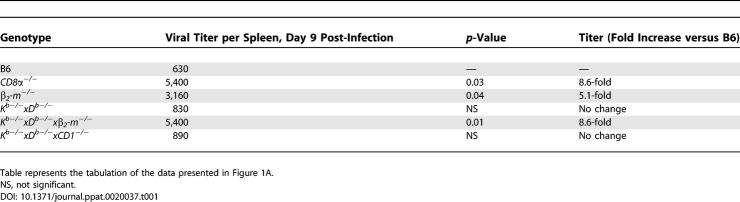
Viral Titer in Spleen 9 Days after Infection

### Class Ia–Deficient Mice Effectively Control Chronic γHV68 Infection

We next evaluated the role of Class Ia molecules in control of chronic γHV68 infection in peritoneal cells and splenocytes from B6, *K^b−/−^xD^b−/−^,* and *K^b−/−^xD^b−/−^xCD1d^−/−^* mice (Figure 1B and 1C, Tables 2 and 3). As read-outs for chronic infection we quantified the frequency of latently infected cells bearing viral genome, the frequency of latently infected cells that reactivated from latency when explanted onto murine embryonic fibroblast (MEF) monolayers, and persistent virus replication [23,25]. Mice lacking Class Ia or both Class Ia and CD1d were able to control chronic γHV68 infection in both the peritoneum and spleen as well as wild-type mice, as measured by either the frequency of cells that reactivate from latency or the frequency of latently infected cells carrying viral genome (Figure 1B and 1C). Thus, as observed for acute infection, mice lacking Class Ia or both Class Ia and CD1d molecules exhibited a significant capacity to control chronic γHV68 infection.

**Table 2:**
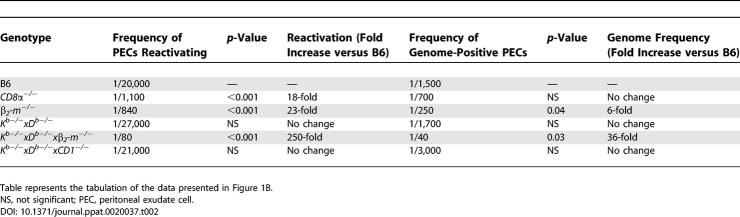
Peritoneal Exudate Cells, Day 42 Post-Infection

**Table 3:**
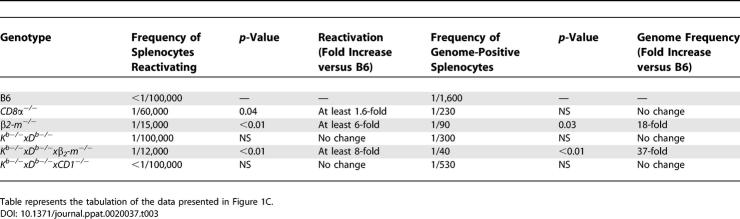
Splenocytes, Day 42 Post-Infection

### Control of Chronic γHV68 Infection in Class Ia–Deficient Mice Is Dependent on β_2_-m and CD8 T Cells

Having shown that MHC Class Ia molecules are not required for control of either acute or chronic γHV68 infection, we next determined if control of chronic γHV68 infection in the absence of Class Ia required β_2_-m or CD8 T cells. B6, β_2_-*m^−/−^, K^b−/−^xD^b−/−^,* and *K^b−/−^xD^b−/−^xβ_2_-m^−/−^* mice were evaluated at 42 d post-infection. For both peritoneal cells and splenocytes, all measures of latent infection in *β_2_-m^−/−^* and *K^b−/−^xD^b−/−^xβ_2_-m^−/−^* mice indicated significant loss of control compared to wild-type and *K^b−/−^xD^b−/−^* mice (Figure 1B and 1C, Tables 2 and 3). Together, these data strongly indicated that control of γHV68 latency and persistent replication is β_2_-m dependent in either the presence or absence of Class Ia molecules.

Since CD8 T cells were important for control of chronic γHV68 infection ([25], and Figure S1), even when Class Ia molecules were not (Figure 1), these data were consistent with the hypothesis that a Class Ia–independent, but β_2_-m–dependent, CD8 T cell response was able to control γHV68 infection. We therefore determined if CD8 T cells were important for control of chronic γHV68 infection in the absence of Class Ia molecules. Since dendritic cells are latently infected with γHV68 [4] and can express CD8α [55–57], we wished to deplete CD8αβ^+^ T cells without altering dendritic cells. Furthermore, we wished to avoid depleting unconventional CD8αα^+^ T cells that develop in mice lacking Class Ia [58]. We therefore selected a depleting antibody specific for CD8β that is not expressed on dendritic cells or *CD8αα^+^* CD8 T cells [55,57,59,60]. *K^b−/−^xD^b−/−^xCD1d^−/−^* mice were either mock treated or treated with a CD8β^+^ cell–depleting antibody during infection. The extent of CD8 T cell depletion was assessed by fluorescence-activated cell sorter (FACS) assay and was found to be equal to or greater than 94% effective in these experiments (Figure S2).

Depletion of CD8β^+^ T cells resulted in loss of control of chronic infection in the Class Ia–deficient mice (Figure 1D and 1E, Tables 4 and 5). Loss of control was especially evident in peritoneal cells in which the majority of latently infected cells are macrophages [1], because the frequency of cells reactivating virus, the frequency of cells positive for viral genome, and the amount of persistent replication were all increased (Figure 1D). These data showed that CD8 T cells were required to control chronic γHV68 infection in mice lacking Class Ia molecules, consistent with the hypothesis that these CD8 T cells were stimulated by a Class Ia–independent mechanism.

**Table 4:**
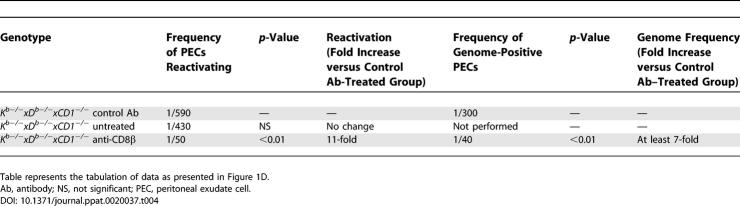
Peritoneal Cells, Day 16 Post-Infection.

**Table 5:**
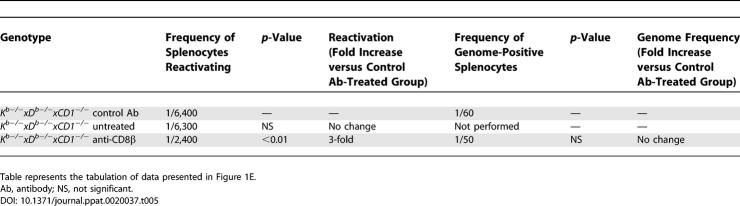
Splenocytes, Day 16 Post-Infection

### Control of Chronic γHV68 Infection in Class Ia–Deficient Mice Is Associated with Stimulation and Expansion of Unconventional CD8 T Cells

**Figure 2:**
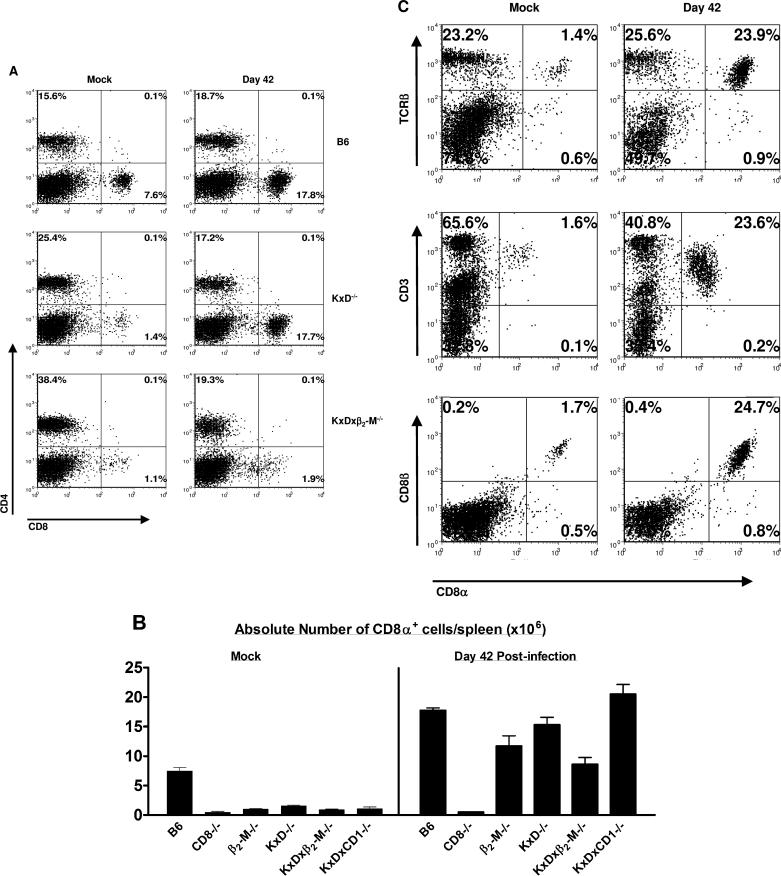
Control of Chronic γHV68 Infection Is Associated with Stimulation of Unconventional CD8 T Cells Splenocytes were harvested from B6, *CD8α^−/−^, β_2_-m^−/−^, K^b−/−^xD^b−/−^, K^b−/−^xD^b−/−^xβ_2_-m^−/−^,* and *K^b−/−^xD^b−/−^xCD1d^−/−^* mice 42 d after mock infection or γHV68 infection and analyzed by flow cytometry. (A) Representative flow cytometric analysis of CD4 and CD8 expression on splenocytes from B6, *K^b−/−^xD^b−/−^,* and *K^b−/−^xD^b−/−^xβ_2_-m^−/−^* mice that were either mock infected or γHV68 infected for 42 d. (B) Absolute number of splenocytes expressing CD8α in mice that were mock infected or infected with γHV68 for 42 d. Data are the mean of four to eight independent experiments using three to five mice per group, ± SEM. (C) Representative flow cytometric analysis of TCRβ, CD3, CD8β, and CD8α expression on splenocytes from B6, *K^b−/−^xD^b−/−^* mice that were either mock infected or γHV68 infected for 42 d.

To more directly assess our hypothesis that CD8 T cells were present in the Class Ia–deficient mice, splenocytes from mock infected and infected B6, *CD8^−/−^, β_2_-m^−/−^, K^b−/−^xD^b−/−^, K^b−/−^xD^b−/−^xCD1d^−/−^,* and *K^b−/−^xD^b−/−^xβ_2_-m^−/−^* mice were characterized for the presence of CD4 and CD8 T cells and for the absolute number and percentage of CD8 T cells. In agreement with previously reported observations, uninfected mice lacking Class Ia molecules (*K^b−/−^xD^b−/−^* and *K^b−/−^xD^b−/−^xCD1d^−/−^* mice) had a significantly lower percentage of CD8 T cells compared to B6 mice (Figure 2A and 2B). This phenotype was even more pronounced in mice lacking both MHC Class Ia and β_2_-m, which was expected because these mice lack expression of all Class Ia and β_2_-m–dependent Class Ib molecules. In infected B6, *K^b−/−^xD^b−/−^,* and *K^b−/−^xD^b−/−^xCD1d^−/−^* mice, however, the percentages and absolute numbers of both CD4 and CD8 T cells were similar (Figure 2A and 2B), indicating that viral infection stimulated a massive expansion of CD8 T cells in mice lacking the Class Ia and CD1d molecules. To confirm that the CD8 T cells observed in the spleens of infected *K^b−/−^xD^b−/−^* mice were T cells, CD8^+^ splenocytes from *K^b−/−^xD^b−/−^* mice were further characterized by flow cytometry and shown to express TCRβ, CD3, and CD8β (Figure 2C). These data demonstrated that the unconventional CD8^+^ cells that expand in *K^b−/−^xD^b−/−^* mice after γHV68 infection were indeed CD8 T cells.

We compared the expansion of CD8 T cells in Class Ia–deficient mice to that observed in mice lacking both Class Ia and β_2_-m (Figure 2A and 2B). There was an increase in CD8 T cells in mice lacking both Class Ia and β_2_-m, and a significant part of the CD8 T cell expansion in Class Ia–deficient mice was β_2_-m dependent. We speculate that the T cells that develop in the absence of both β_2_-m and Class I may be related to cells previously shown to respond to γHV68 infection in the absence of β_2_-m [42,44].

### Unconventional CD8 T Cells Induced by γHV68 Infection in Class Ia–Deficient Mice Demonstrate an Activated Phenotype

**Figure 3:**
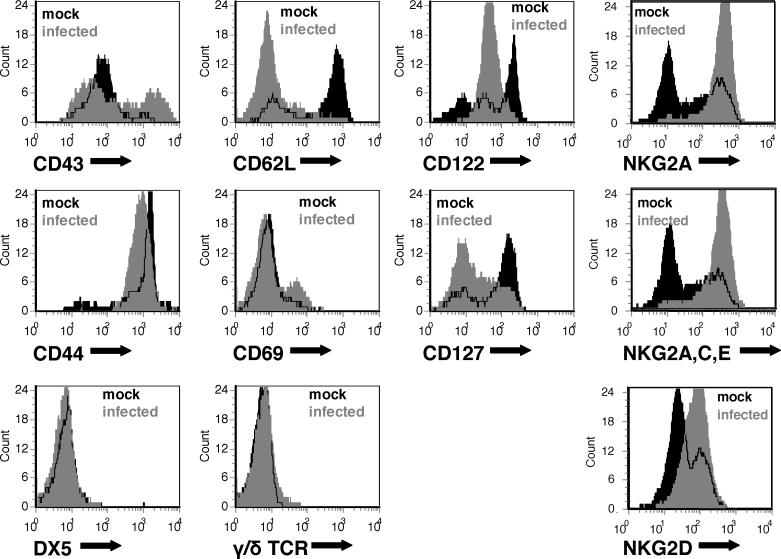
CD8 T Cells Induced by γHV68 Infection in Class Ia–Deficient Mice Demonstrate an Activated Phenotype Splenocytes were harvested from *K^b−/−^xD^b−/−^* mice 42 d after mock infection or γHV68 infection. CD8α^+^ splenocytes from these mice were analyzed by flow cytometry for cell surface expression of markers of effector/memory phenotype and expression of NK and T cell markers. Panels are representative of the flow cytometric analysis from two independent experiments.

We further evaluated the CD8 T cells that expand during infection of Class Ia–deficient mice by determining whether they express cell surface markers observed on conventional Class Ia–dependent effector/memory CD8 T cells. Using flow cytometry, we compared expression of cell surface markers between mock-infected Class Ia–deficient mice and mice that had been infected for 42 d (Figure 3). The results were consistent with the cells being effector/memory cells (CD43^+^, CD69^+/−^, CD62L-low, CD122^+/−^ [IL2Rβ chain], CD127-low [IL7Rα chain]) [61–63]. We also evaluated the cells for the pan–natural killer (NK) cell surface marker DX5 (CD49b) and antibodies specific for the γδT cell receptor; all of which were negative on both mock-infected and infected unconventional CD8 T cells. Finally, the cells were evaluated for expression of known CD8 T cell–associated NK cell markers characteristic of activated cytotoxic CD8 T cells [63]. The unconventional CD8 T cells were highly positive for NKG2A and expressed elevated amounts of NKG2D. Together, these results were consistent with the unconventional CD8 T cells being effector/memory cells and, therefore, with the cells being anti-viral T cells.

### Unconventional CD8 T Cells Are Vβ4-Biased after γHV68 Infection

**Figure 4:**
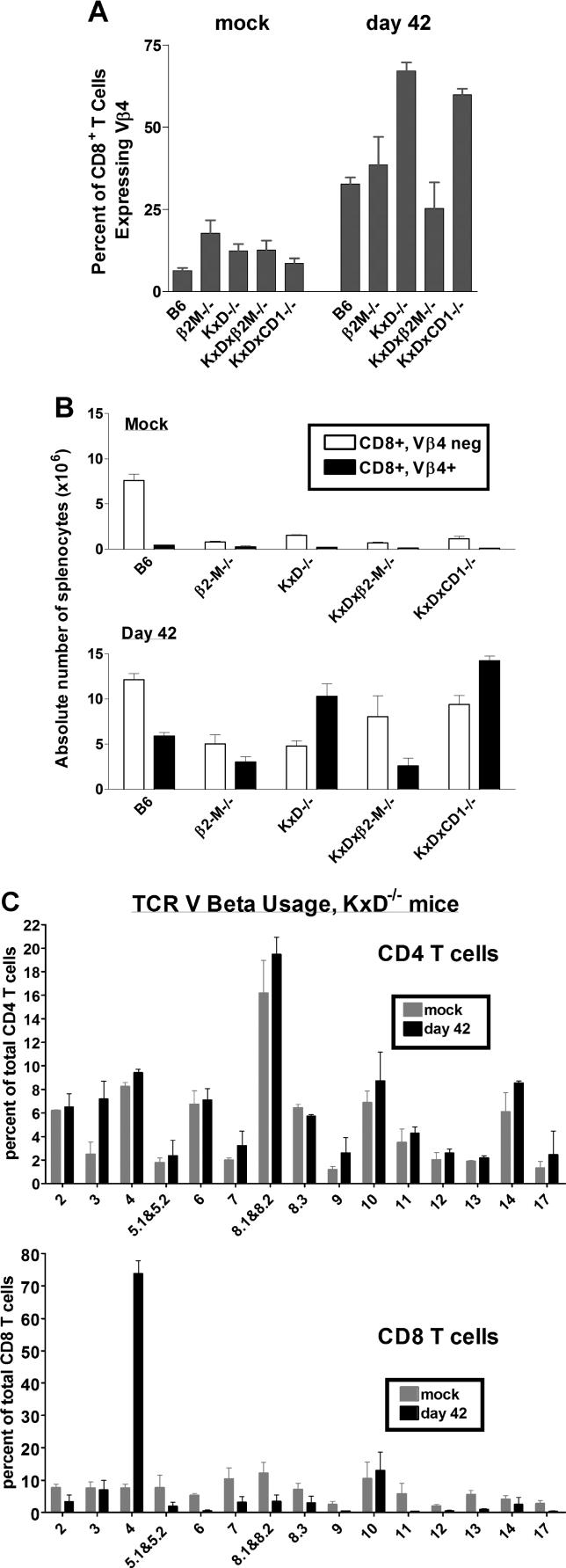
CD8 T Cells from γHV68-Infected Class Ia–Deficient Mice Are Vβ4 Biased Splenocytes were harvested from B6, β_2_-*m^−/−^, K^b−/−^xD^b−/−^, K^b−/−^xD^b−/−^xβ_2_-m^−/−^,* and *K^b−/−^xD^b−/−^xCD1d^−/−^* mice 42 d after mock infection or γHV68 infection. TCR Vβ usage by CD4 and CD8 T cells from these mice was assessed by flow cytometry. (A) Percentage of total CD8α^+^ splenocytes that co-express Vβ4. Data are the mean of four to eight independent experiments using three to five mice per group, ± SEM. (B) Absolute number of *CD8α^+^Vβ4^−^* and *CD8α^+^Vβ4^+^* splenocytes. Data are the mean of four to eight independent experiments using three to five mice per group, ± SEM. (C) TCR Vβ analysis of CD4 and CD8 T cells obtained from the spleens of *K^b−/−^xD^b−/−^* mice 42 d after mock-infection or γHV68 infection. Data are the mean ± SEM of three independent experiments.

In wild-type, *β_2_-m^−/−^,* and *TAP1^−/−^* mice, the CD8 T cell response to γHV68 is Vβ4 biased [26,42]. We therefore used flow cytometry to characterize Vβ usage in infected versus control B6, *K^b−/−^xD^b−/−^,* and *K^b−/−^xD^b−/−^xβ_2_-m^−/−^* mice (Figure 4A and 4B). For CD4 T cells, Vβ usage was not significantly skewed by γHV68 infection; for CD8 T cells, Vβ usage was predominantly Vβ4 biased after infection. This contrasts with uninfected Class Ia–deficient mice, which have previously been shown not to have biased Vβ TCR usage compared to wild-type mice [64]. The percentage of total splenic CD8 T cells expressing Vβ4 increasing from 6% to approximately 40% by 42 d after infection (Figure 4A). In infected *K^b−/−^xD^b−/−^* mice, the percentage of Vβ4 T cells (about 70%) was much higher than that observed in B6 mice. These results confirmed that Class Ia molecules are not required for stimulating the Vβ4 T cells [42] and that the majority of CD8 T cells in Class Ia–deficient mice were biased toward Vβ4 usage. In infected *K^b−/−^xD^b−/−^xβ_2_-m^−/−^* mice, however, the percentage of CD8 T cells that express Vβ4 was significantly less (*p* < 0.01) than that seen in *K^b−/−^xD^b−/−^* mice (Figure 4A), which was consistent with the stimulation of Vβ4 CD8 T cells in *K^b−/−^xD^b−/−^* mice being significantly but not completely dependent on β_2_-m.

In addition to the increase in Vβ4 usage, we observed that both Vβ3 and Vβ10 usage were maintained in infected as compared to uninfected Class Ia–deficient mice (Figure 4C). Given the large increase in total CD8 T cells stimulated by γHV68 infection, this indicated that both Vβ3- and Vβ10-expressing T cells expanded in infected Class Ia–deficient mice, although to a lesser extent than Vβ4-expressing T cells. Together these data demonstrated significant Vβ bias in CD8 T cells that develop during chronic γHV68 infection of Class Ia–deficient mice.

### T Cell Receptor Diversity in Unconventional CD8 T Cells that Control Chronic γHV68 Infection

The above bias could be due to a very limited repertoire of T cells selected on and restricted by Class Ib MHC molecules; in which case only a few clones could account for the expansion of Vβ4 and other families. To assess clonal complexity of unconventional CD8 T cells that develop during γHV68 infection, we compared CDR3 length complexity in CD8 T cells from infected and uninfected mice. CDR3 regions are the most polymorphic parts of the TCR, and their diversity is representative of T cell diversity within a given population. CDR3 length analysis determines in a population of cells the relative abundance of mRNAs encoding TCRs with specific CDR3 lengths (usually between 18–39 base pairs (bp) encoding CDR3 regions of 6–13 amino acids) [65]. In young, non-immunized mice, this analysis reveals within each TCR Vβ family a pattern of CDR3 length peaks spaced 3 bp apart in a Gaussian distribution around the most frequent length (27–30 bp encoding 9–10 amino acids), representative of a diverse T cell repertoire [65]. Antigenic challenge induces expansion of a handful of Ag-specific T cell clones, leading to relative or absolute dominance of one (monoclonal response) or a few (oligoclonal response) peaks in the profile [66,67]. During the contraction phase of the T cell response, expanded T cell clones die by apoptosis, restoring the Gaussian CDR3 length profile. Therefore, distorted profiles, with a few, or only a single, peak, are strongly suggestive of restricted TCR utilization, whereas a Gaussian distribution of CDR3 lengths within proliferating T cells is indicative of diverse TCR utilization.

**Figure 5:**
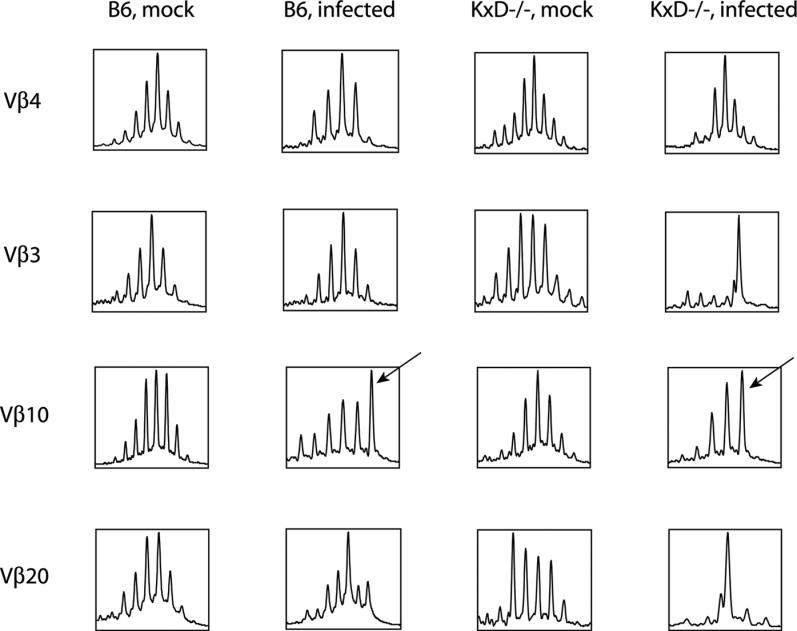
The Anti-γHV68 CD8 T Cell Response Encompasses Substantial CDR3 Length Diversity in Both Wild-Type and *K^b−/−^xD^b−/−^* Mice TCR CDR3 length analysis of CD8 T cells obtained from the spleens of B6 and *K^b−/−^xD^b−/−^* mice 42 d after mock-infection or γHV68 infection. Shown on the *x*-axis is CDR3 length within the CD8 T cell populations expressing Vβ3, Vβ4, Vβ10, or Vβ20. The *y*-axis represents the relative abundance of mRNA encoding TCR chains of individual CDR3 lengths. Data are from one of two representative experiments. Arrows indicate an asymmetric peak to the right of the center observed in the subdominant Vβ10 response from both the wild-type and *K^b−/−^xD^b−/−^* mice.

CDR3 length analysis revealed that the CD8 T cells from a broad range of Vβ families retained CDR3 length diversity in γHV68-infected wild-type mice (unpublished data and Figure 5), suggesting that numerous T cell clones from a variety of Vβ families respond to chronic γHV68 infection. This included the dominant Vβ4^+^ population of T cells that showed signs of polyclonal response (Figure 5). In contrast, the CDR3 length profiles of unconventional CD8 T cells that developed in infected *K^b−/−^xD^b−/−^* mice revealed two patterns (Figure 5, and unpublished data). The dominant Vβ4 and subdominant Vβ10 families, comprising more than 80% of the total CD8 response of *K^b−/−^xD^b−/−^* mice, displayed diversity similar to that seen in wild-type mice. This suggests that much of the response in both wild-type and MHC Class Ia–deficient mice to γHV68 is diverse. Of note, the subdominant Vβ10 response in both the wild-type and *K^b−/−^xD^b−/−^* mice included an asymmetric peak to the right of the center (Figure 5, arrows), suggesting that the response in normal animals may include Class Ia–independent clones. A second pattern was seen in the case of Vβ3 and Vβ20 TCR families, where peaks suggestive of oligoclonal or monoclonal expansions are seen in Class Ia–deficient, but not wild-type, animals (Figure 5). Sequencing analysis is in progress to ascertain that some of the Class Ia–independent clones are invoked in the response of wild-type animals. Together, these data indicated that the Class Ia–independent anti-γHV68 CD8 T cell response encompasses substantial diversity, indicating that the development of these cells is not restricted to a limited and highly specialized subset of T cells.

### Unconventional CD8 T Cells Express Effector Molecules in Infected Class Ia–Deficient Mice

Because unconventional CD8 T cells were capable of controlling γHV68 infection in Class Ia–deficient mice (Figure 1), we examined their capacity to express effector molecules known to be important for control of chronic γHV68 infection. We focused on expression of IFNγ, which is of proven importance for control of latent γHV68 infection, reactivation of γHV68 from latency, and persistent γHV68 replication [15,16,25,31–33]. The unconventional CD8 T cells in splenocytes from *K^b−/−^xD^b−/−^* mice were compared to CD8 T cells in splenocytes from B6 mice. Detection of the expression of cytokines by effector CD8 T cells in other viral systems often requires re-stimulation of cells in vitro. As we do not know the antigen specificity of the unconventional CD8 T cells that develop in γHV68-infected Class Ia–deficient mice, we restimulated cells with phorbol myristate acetate (PMA) and ionomycin prior to staining for expression of IFNγ and tumor necrosis factor-α (TNFα). Unstimulated cells expressed only low levels of IFNγ and TNFα in these experiments (unpublished data).

**Figure 6:**
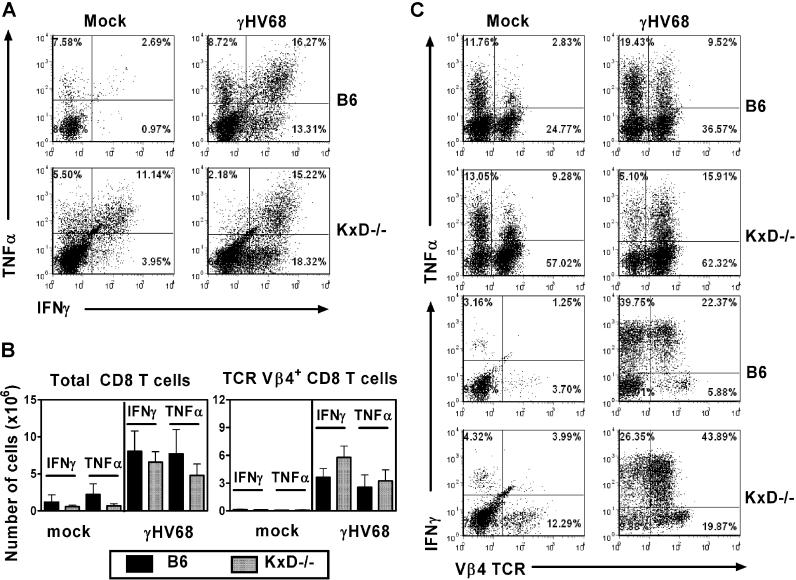
Unconventional CD8 T Cells Express Effector Molecules in Infected Class Ia–Deficient Mice Splenocytes were harvested from B6 and *K^b−/−^xD^b−/−^* mice 42 d after mock infection or γHV68 infection, and analyzed by flow cytometry. (A) Representative flow cytometric analysis of intracellular IFNγ and TNFα expression by CD8 T cells from B6 and *K^b−/−^xD^b−/−^* mice that were either mock infected or γHV68 infected for 42 d. (B) Absolute number of total CD8 T cells and Vβ4 CD8 T cells expressing intracellular IFNγ and TNFα in mice that were mock infected or γHV68 infected for 42 d. Data are the mean of two independent experiments using five to ten mice per group, ± SEM. (C) Representative flow cytometric analysis of intracellular IFNγ, intracellular TNFα versus Vβ4 expression by CD8 T cells from B6 and *K^b−/−^xD^b−/−^* mice that were either mock-infected or γHV68-infected for 42 d. Representative from one of two experiments.

CD8 T cells from mock-infectedB6 mice produced little TNFα and no IFNγ even after stimulation with PMA and ionomycin. In contrast, approximately 11% of CD8 T cells from uninfected *K^b−/−^xD^b−/−^* mice produced both TNFα and IFNγ after PMA and ionomycin stimulation, whereas smaller populations of cells produced either TNFα or IFNγ alone (Figure 6A). These data demonstrated a functional difference between unstimulated conventional and unconventional CD8 T cells, and indicate that even in naive *K^b−/−^xD^b−/−^* mice, the CD8 T cells were in a partially activated state. These findings are consistent with previous data obtained in characterizing these cells [58,68].

In B6 mice 42 d after infection, PMA and ionomycin treatment induced a significant percentage of CD8 T cells to express IFNγ, TNFα, or both cytokines (Figure 6A). Similarly, infection of *K^b−/−^xD^b−/−^* mice resulted in a significant increase in the proportion of PMA and ionomycin–stimulated cells that expressed IFNγ (Figure 6A). Because the majority of CD8 T cells that develop in γHV68-infected Class Ia–deficient mice express Vβ4, we also examined cytokine expression in Vβ4 CD8 T cells (Figure 6B and 6C). In both wild-type and Class Ia–deficient mice, γHV68 infection induced a significant increase in CD8 T cells expressing IFNγ (Figure 6B and 6C). It would be of interest to examine these cells for the expression of IFNαβ, which also can control γHV68 latency [33]. These data were consistent with CD8 T cells in both B6 and *K^b−/−^xD^b−/−^* mice acquiring an activated phenotype after γHV68 infection, confirming our conclusions from analysis of cell surface molecule expression on these cells (Figure 3). Because IFNγ is important for control of γHV68 latency and persistent replication, these data indicated that γHV68-induced CD8 T cells that develop in Class Ia–deficient mice express important anti-viral effector molecules.

## Discussion

We present data in this paper demonstrating that unconventional CD8 T cells arise in infected Class Ia–deficient mice. We show furthermore that these unconventional CD8 T cells express surface markers and cytokines consistent with memory and effector function and that they are required for control of chronic γHV68 infection in the absence of classical Class Ia molecules. The unconventional CD8 T cells are β_2_-m–dependent, but CD1d-independent, and predominantly express Vβ4, Vβ3, and Vβ10, as well as NK cell markers such as NKG2A and NKG2D. They exhibit a diverse TCR repertoire, consistent with a potential to respond to different viral antigens. This is the first demonstration of the development of a diverse and functionally important Class Ia–independent CD8 T cell response during chronic viral infection.

Several previously published studies have demonstrated that CD8 T cells are important for control of γHV68 infection [19,20,25,43], and that Class Ia–restricted CD8 T cells specific for both lytic and latent γHV68 antigens can mediate protection [21,39]. Furthermore, vaccination with immunodominant Class Ia–binding γHV68 peptides can decrease acute titers and transiently decrease the early form of latency [69–71], and adoptive transfer of latent virus antigen-specific Class Ia–restricted CD8 T cells decreases latency early after infection [28]. Thus, there is no question that conventional Class Ia–restricted CD8 T cells are an important part of the response to γHV68 infection. The data presented here add unconventional CD8 T cells to the host's anti-viral armamentarium for control of chronic infection, and indicate that the role of unconventional CD8 T cells needs to be systematically explored in chronic viral infections. These T cells might be particularly important for control of viruses that actively inhibit expression of host cell MHC Class I as a mechanism of immune evasion.

Our experiments did not address the extent to which unconventional CD8 T cells function as effector cells in wild-type mice. We speculate that these cells do play a role in normal hosts based on the presence of T cells with similar markers in normal hosts and the diverse TCR repertoire used by these unconventional CD8 T cells. Moreover, CDR3 repertoire analysis suggests that some CDR3 lengths (and, perhaps, T cell clones) may be shared between the responses in wild-type and *K^b−/−^xD^b−/−^* mice. However, the proof that such cells play a role when Class Ia molecules are present must await identification of specific mechanisms of activation and selection for these cells and loss of function approaches based on that information, or identification of cell surface molecules that are specific to this interesting set of CD8 T cells, followed by depletion studies.

The observation that β_2_-m deficiency disrupts immunity to chronic γHV68 infection was initially attributed to decreased Class Ia expression on β_2_-m–deficient cells [40]. This interpretation is not correct because data presented here demonstrate that effective control of long-term latency does not require Class Ia molecules. Moreover, since β_2_-m deficiency resulted in a more-severe loss of control of latency than did Class Ia deficiency alone, we hypothesize that β_2_-m has an important functional role in controlling infection in addition to its more commonly known function as the essential light chain for Class Ia proteins. Consistent with this, *K^b−/−^xD^b−/−^xβ_2_-m^−/−^* mice have much higher levels of latency and persistent infection in both splenocytes and peritoneal cells than *K^b−/−^xD^b−/−^* mice. The simplest explanation for this Class Ia–independent role for β_2_-m in control of chronic viral infection is that β_2_-m–dependent, but Class Ia–independent, unconventional CD8 T cells are important for control of γHV68 infection. We speculate that these cells are restricted by β_2_-m–dependent MHC Class Ib molecules.

T cells restricted to MHC Class Ib proteins are often mono- or oligoclonal with regard to TCRα and/or TCRβ usage. This characteristic has been observed in HLA-E-restricted CD8 T cells in humans [72], MR1-restricted mucosal-associated T cells [73], and CD1d-restricted NKT cells [74]. Thus, the Vβ4-, Vβ3-, and Vβ10-biased nature of the CD8 T cell response to γHV68 in Class Ia–deficient mice is consistent with the presence of one or more populations of MHC Class Ib–restricted CD8 T cells. An important role for one or more MHC Class Ib molecules in γHV68 immunity could therefore explain both the importance of β_2_-m for efficient expansion of CD8 T cells in Class Ia–deficient mice, and the Vβ bias of the unconventional CD8 T cells demonstrated herein.

We ruled out a contribution for CD1d in control of chronic γHV68 infection, but other candidates remain. CD8 T cells restricted to the Class Ib molecules Qa-1 and H2-M3 contribute to resistance to intracellular bacteria such as Listeria monocytogenes and Mycobacterium tuberculosis [75–78]. In humans, H2-M3 has been shown to present influenza virus peptides to a human CD8 T cell clone [79]. In addition, a population of CD8 T cells that recognizes peptides derived from human beta- and γ-herpesviruses in the context of HLA-E, the human homolog of Qa-1, has been identified [72]. This latter observation is of particular interest since the CD8 T cells that developed during chronic γHV68 infection express high levels of NKG2A, which interacts with Qa1.

In summary, we demonstrate here that γHV68 infection triggers a robust β_2_-m–dependent, Class Ia–independent, and CD1d-independent unconventional CD8 T cell response that can control γHV68 latency and persistent replication. These findings make two important points regarding immunity to chronic viral infection: (1) Class Ia–independent non-classical CD8 T cells can control latent herpesvirus infection, and (2) the role of β_2_-m in immunity to chronic viral infection is not limited to its function as a light chain for Class Ia molecules. It is possible that similar Class Ia–independent CD8 T cells are required for control of other chronic viral infections, such as human herpesviruses, HIV, hepatitis C virus, hepatitis B virus, and lymphocytic choriomeningitis virus (LCMV). Furthermore, since Class Ia proteins are not essential for anti-viral CD8 T cell responses, CD8 T cell–directed vaccination strategies that target only Class Ia–presented antigens may not induce a maximal antiviral response.

## Materials and Methods

### Mice.

*C57BL/6J* [B6, Jackson# 000664] mice and mice deficient in either the CD8α chain (*CD8^−/−^,* Jackson# 002665, [80]) or β_2_-m (*β_2_-m^−/−^,* Jackson# 002087, [81,82]) were obtained from The Jackson Laboratories (Bar Harbor, Maine, United States). *K^b−/−^xD^b−/−^* mice [51] were a generous gift from both Dr. Ted Hansen and Dr. Albert Bendelac. *K^b−/−^xD^b−/−^x β_2_-m^−/−^ (K^b−/−^xD^b−/−^ x β_2_-m^−/−^)* mice were a generous gift of Dr. Ted Hansen [52]. *CD1d^−/−^* [83] and *K^b−/−^xD^b−/−^xCD1d^−/−^ (K^b−/−^xD^b−/−^x CD1d^−/−^)* mice were a generous gift of Dr. Albert Bendelac. All mice were on the B6 background and were bred and housed at Washington University School of Medicine in accordance with all Federal and University guidelines. Eight- to 12-wk-old mice were used for all experiments.

### Cell culture and virus infection.

NIH 3T12 and B6 mouse embryonic fibroblasts were maintained as described [40]. γHV68 clone WUMS (ATCC VR1465) was passed and titered by plaque assay on NIH 3T12 cells [40]. Mice were infected intraperitoneally with 10^6^ PFU in 0.5 ml Dulbecco modified Eagle's Medium with 1% fetal calf serum (DMEM-1) [23]. Mock-infected animals were injected intraperitoneally with NIH 3T12 cell lysate diluted in DMEM-1.

### Preparation of splenocytes, antibodies, and flow cytometry.

Single-cell suspensions of splenocytes were prepared for flow cytometry as described [40]. Splenocytes, 10^6^ per condition, were incubated in FACS-blocking buffer (1× PBS with 1.0% BSA, 0.1% sodium azide plus rat anti-mouse CD16/CD32 [clone 2.4G2, ATCC#HB-197] and 5% rat serum) for 30 min on ice. Five percent mouse serum was added for NKG2A/C/E staining. Antibody staining for cell surface markers was performed in 100 μl of FACS buffer (1× PBS with 1.0% BSA, 0.1% sodium azide) on ice for 45 min, followed by three washes of (200 μl each) of FACS buffer. The cells were then fixed in 1% paraformaldehyde (in PBS) and stored in FACS buffer until analyzed. For intracellular staining, cells were activated with 20-ng/ml PMA plus1 μM ionomycin for 4–6 h in the presence of GolgiPlug from the Cytofix/Cytoperm kit from BD Pharmingen (San Diego, California, United States). After staining the cells for surface markers, cells were fixed and permeabilized using the Cytofix/Cytoperm kit according to the manufacturer's instructions. Next, the cells were stained with antibodies specific to IFNγ and TNFα, and then washed and resuspended in FACS buffer prior to analysis. PE anti-CD43, CD62L, CD122, CD127, CD44, CD69, DX5, CD4, αβTCR, TNFα, PE-labeled strepavidin, CyChrome anti-CD3, fluorescein isothiocyanate (FITC) anti-CD4, PE, allophycocyanin (APC) and Alexa 488 anti- IFNγ, biotin anti-NKG2A/C/E, and FITC anti-TCRβ (pan β, clone H57–597) were obtained from BD Pharmingen. TCR V Beta usage of T cells from mock-infected and infected *K^b−/−^xD^b−/−^* mice was performed using the mouse Vβ TCR Screening Panel (BD Pharmingen, Catalog# 557004) according to the manufacturer's instructions. Labeled isotype–matched control antibodies were obtained from BD Pharmingen. Stained cell populations were processed with a FACSCaliber flow cytometer (BD Pharmingen), and the data were analyzed with either CellQuest (BD Pharmingen) or FCS Express Version 2 software (De Novo Software, Ontario, Canada).

### CDR3 length analysis.

The PCR conditions and the primers were described previously [84]. CDR3 length polymorphism profiles of Vβ families were obtained using the Immunoscope software, depicting the relative intensity of bands migrating to the same CDR3 nucleotide length, as previously described [84].

### Plaque assays.

Plaque assays were performed as previously described [40]. Briefly, organs were harvested into sterile, screw-top 2-ml tubes containing 1 ml of DMEM and 100 μl of 1-mm zirconia/silica beads (BioSpec Products, Bartlesville, Oklahoma, United States) and stored at −80 °C. Samples were thawed on ice and homogenized using a Mini BeadBeater (BioSpec Products), then further diluted in DMEM prior to infecting NIH 3T12 monolayers. Infected monolayers were overlaid with Noble agar, and plaques were visualized at 7 d post-harvest using neutral red staining. The limit of detection was 50 PFU.

### In vivo depletion of lymphocyte subsets.

Monoclonal antibodies were produced in INTEGRA Celline CL1000 flasks (Integra Biosciences, Ijamsville, Maryland, United States) as described [20]. Beginning 1 d prior to virus infection, 2 mg of CD8-depleting antibody (H35, [85]) or an isotype-matched control antibody (SFR3-DR5, IgG2b, ATCC HB-151, [86]) were administered to each mouse by intraperitoneal injection. Monoclonal antibodies (1 mg) were administered every fourth day thereafter. The efficacy of CD8 depletion was monitored by flow cytometric analysis of splenocytes and was 94% or greater in each case.

### Ex vivo limiting dilution reactivation analysis.

The frequency of cells reactivating from latency was assayed as described [40]. Briefly, serial 2-fold dilutions of harvested cells (24 wells/dilution starting at 1 × 10^5^ cells/well for splenocytes and 4 × 10^4^ cells/well for peritoneal cells) were plated onto permissive mouse embryonic fibroblast monolayers (MEFs) for 21 d, then scored for cytopathic effect that are due to reactivating virus. To determine if samples contained preformed infectious virus at harvest due to persistent replication, replicate cell aliquots were mechanically disrupted prior to limiting dilution and plating.

### Limiting dilution PCR analysis.

To determine the frequency of cells carrying the γHV68 genome, single-copy sensitivity nested PCR for the γHV68 *v-cyclin (gene 72),* was performed on serial dilutions of cells as described [1,24]. Primers for *v-cyclin* were 5′gagatctgtactcaggcacctgt3′ and 5′ggatttcttgacagctccctgt3′ for round 1, and 5′tgtcagctgttgttgctcct3′ and 5′ctccgtcaggataacaacgtct3′ for round 2. One false positive was detected in 246 reactions across all experiments. Positive control reactions containing ten, one, or 0.1 copies of *v-cyclin* plasmid DNA were positive in 99%, 42%, and 5% of all reactions, respectively.

### Statistical analysis.

All data points represent the mean ± the standard error of the mean (SEM) for two to three experiments with three to five mice per condition per experiment. To quantify the frequency of cells from which the virus reactivated or that carried latent viral genome, data were subjected to nonlinear regression using GraphPad Prism (GraphPad, San Diego, California, United States). Frequencies were determined using the Poisson distribution assuming that the cell number at which 63.2% of the wells scored positive for reactivation or viral genome represented a single event. To calculate significance, data were statistically analyzed by unpaired *t* test. For limiting dilution analyses, the calculated frequencies were compared by unpaired *t* test.

## Supporting Information

Figure S1 Control of Chronic γHV68 Infection Is Dependent on CD8 T Cells (A) Frequency of cells reactivating from latency ex vivo (left), frequency of cells bearing viral genome (middle), and persistent replication (right) at day 42 post-infection in peritoneal cells from B6, *K^b−/−^xD^b−/−^,* and *CD8^−/−^* mice. On the *y*-axis is the percentage of wells positive for viral cytopathic effect (left and right) or viral genome (middle). The horizontal line within the graph indicates the 63.2% Poisson distribution line used to calculate the frequency of cells reactivating virus. Data are the mean of three to four independent experiments ± SEM.
(B) Frequency of latent infection in splenocytes from B6, *K^b−/−^xD^b−/−^,* and *CD8^−/−^* mice. Data are the mean of three to four independent experiments ± SEM.
(219 KB TIF)

Figure S2 Efficacy of CD8 T Cell Depletion as Assessed by Flow Cytometry Splenocytes from *K^b−/−^xD^b−/−^xCD1d^−/−^* mice that were either treated with a CD8-depleting antibody (Ab) or an irrelevant control antibody were harvested 16 d after γHV68 infection and analyzed by flow cytometry. Panels show representative flow cytometric analysis of CD4 and CD8 expression on splenocytes.
(398 KB TIF)

## Abbreviations

γHV68: *γ-herpesvirus 68*
bp: base pair
FACS: fluorescence-activated cell sorter
IFNγ: interferon-γ
MHC: major histocompatibility complex
NK: natural killer
PFU: plaque forming unit
PMA: phorbol myristate acetate
SEM: standard error of the mean
TNFα: tumor necrosis factor-α
β2-m: β2-microglobulin
